# Psychological Impact in Individuals with Monoclonal Gammopathy of Undetermined Significance and Smoldering Multiple Myeloma

**DOI:** 10.46989/001c.123608

**Published:** 2024-09-24

**Authors:** Tanvi H. Patel, Ramya Bachu, Trilok Shrivastava, Jawad Alrawabdeh, Marah Alzubi, Jael Hastings, Harold Dean, Carolina Schinke, Sharmilan Thanendrarajan, Maurizio Zangari, Guido Tricot, Fenghuang Zhan, John D Shaughnessy, Frits van Rhee, Samer Al Hadidi

**Affiliations:** 1 Hematology and Oncology University of Arkansas for Medical Sciences; 2 Hematology and Oncology University of Arkansas for Medical Sciences; 3 Medicine University of Jordan

**Keywords:** Multiple myeloma, monoclonal gammopathy of undetermined significance, smoldering multiple myeloma, psychological effects, anxiety

## Abstract

In our study of 246 newly diagnosed individuals with MGUS or SMM (115 MGUS, 131 SMM), we found that 19% reported anxiety, with no significant difference between the MGUS and SMM groups (22% vs. 17%). Those with a history of psychiatric disorders or belonging to certain racial groups were more likely to experience anxiety. Initial coping responses included religious coping, denial, frustration, irritability, and seeking social support. Given anxiety’s detrimental effects, our findings emphasize the importance of incorporating psychosocial assessments to optimize care for MGUS and SMM patients.

## Introduction

Monoclonal gammopathy of undetermined significance (MGUS) and smoldering multiple myeloma (SMM) are medical conditions characterized by asymptomatic clonal plasma cell process that have the potential to develop into multiple myeloma (MM).^1,2^ In a population-based study, the prevalence of MGUS was determined to be 3.2% in individuals aged 50 or older.^3^ The prevalence increased with age, reaching 5.3% in those aged 70 or older. Whereas, a study found the prevalence of SMM to be 0.5% in individuals aged 40 or older.^4^ SMM is associated with higher risk of progression to MM with 59% of patients developing MM during a 26-year period.^5^ The diagnosis of MGUS and/or SMM may be linked to psychological effects that can have a detrimental impact on patients’ quality of life (QoL).^6^ A previous study revealed that MGUS patients reported significantly lower QoL compared to SMM patients, experiencing a heightened sense of loss of control over their multiple myeloma (MM) risk.^7^ MGUS and SMM are life-long conditions that may or may not advance to MM and in which surveillance is essential, contributing to uncertainty and fear. Our study aimed to evaluate the prevalence of adverse psychosocial effects among individuals with newly diagnosed MGUS and/or SMM.

## Patients and Methods

From August 2015 to February 2023, we conducted prospective interviews with all patients referred to the University of Arkansas for Medical Sciences who had newly diagnosed MGUS and SMM before their consultation with the oncologist as part of our standard clinical care at our institution. Professionally trained social workers systematically conducted an initial psychosocial assessment, employing a comprehensive approach to evaluate the multifaceted dimensions of patients’ psychological, emotional, social, and familial well-being. The purpose of this assessment was to attain a nuanced understanding of each patient’s psychosocial status. Subsequently, the social workers diligently transcribed their findings, observations, and insights into comprehensive narrative reports for every patient referred during the specified period. Two authors (T.P. and R.B.) collected demographic and clinical variables from patients’ electronic medical records Anxiety was reported by patients at time of interview and does not necessarily mean they were diagnosed with anxiety disorder based on clinical criteria.

## Statistical Analysis

Descriptive variables underwent frequency-based statistical analysis. Associations were assessed using Pearson’s chi-square and Fisher’s exact tests. Univariable logistic regression identified anxiety risk factors in MGUS and SMM patients. Subsequently, variables demonstrating statistical significance (p < 0.05) were incorporated into a multivariable logistic regression model. Additionally, thematic analysis was conducted to identify recurring patterns or themes in the collected quotations, with two authors achieving consensus on all established themes. The IBM SPSS Statistics 28 software was utilized for data analysis. The University of Arkansas for Medical Sciences institutional review board approved all study procedures, and a waiver of informed consent was granted due to the retrospective nature of the analysis.

## Results

Our study consisted of 246 patients, with 115 having MGUS and 131 having SMM. The average age was 68 years, and 51% were male, with 81% identifying as non-Hispanic Caucasians. Most patients (91%) were diagnosed incidentally through blood and/or urine tests, and 62% had a family history of cancer. **[Table.1]**

**Table 1.** General Characteristics on included patients (n=246)

	MGUS (n=115)		SMM (n=131)	
Categorical	Count	Percentage	Count	Percentage
Sex	(Missing = 0)		(Missing = 0)	
Male Female	51	44.3	75	57.3
	64	55.7	56	42.7
Race	(Missing = 2)		(Missing = 7)	
White	91	80.5	107	86.3
African American	20	17.7	16	12.9
Other	2	1.8	1	0.8
Symptoms Based Diagnosis	(Missing = 0)		(Missing = 0)	
Yes No	35	30.4	26	19.8
	80	69.6	105	80.2
Blood/Urine Analysis Diagnosis	(Missing = 0)		(Missing = 0)	
Yes No	99	86.1	124	94.7
	16	13.9	7	5.3
Imaging Based Diagnosis	(Missing = 0)		(Missing = 0)	
Yes No	4	3.5	2	1.5
	111	96.5	129	98.5
Bone Marrow Biopsy Prior to referral	(Missing = 0)		(Missing = 0)	
Yes No	6	5.2	12	9.2
	109	94.8	119	90.8
Social Support	(Missing = 0)		(Missing = 0)	
Yes No	114	99.1	131	100
	1	0.9	0	0
Presence of Anxiety	(Missing = 2)		(Missing = 1)	
Yes No	25	22.1	22	16.9
	88	77.9	108	83.1
History of Psychiatric Disorders	(Missing = 0)		(Missing = 3)	
Yes No	44	38.3	39	30.5
	71	61.7	89	69.5
Alcohol / Drug Usage	(Missing = 0)		(Missing = 0)	
Yes No	21	18.3	16	12.2
	94	81.7	115	87.8
Exposure to Chemicals	(Missing = 22)		(Missing = 29)	
Yes No	31	33.3	45	44.1
	62	66.7	57	55.9
Family History of Cancer	(Missing = 28)		(Missing = 21)	
Yes No	68	78.2	84	76.4
	19	21.8	26	23.6

The mean M protein levels were found to be 0.8 (standard deviation (SD): 0.6) in MGUS and 1.5 (SD:0.9) in SMM. Regarding the involved light chains, Kappa Free Light Chains (KFLC) displayed mean values of 5.4 (SD: 9.6) in MGUS and 12.9 (SD: 19.64) in SMM. Similarly, Lambda Free Light Chains (LFLC) showed mean values of 4.3 (SD: 7.9) in MGUS and 6.0 (SD: 15.2) in SMM. The average age of patients was relatively similar for both MGUS (68.6, SD: 11.7) and SMM (68.1, SD: 11.3) groups, with no significant age-related differences observed. Details on clinical characteristics of initial diagnosis were summarized in **Table.2**

**Table 2.** Clinical characteristics on included patients (n=246)

	MGUS		SMM	
Variable	Mean	± Standard Deviation	Mean	± Standard Deviation
M Protein	.8349	.652	1.5226	.924
KFLC	5.4	9.596	12.876	19.641
LFLC	4.2745	7.869	6.025	15.182
Age	68.58	11.646	68.08	11.33
Plasma Cells in Biopsy (Count, %) <=10% 11-20% 21-30% 31-40% 41-50% 51-60%	103 10 1 1 0 0	89.6 8.7 0.9 0.9 0 0	31 74 13 6 5 1	23.7 56.5 9.9 4.6 3.8 0.8
Plasma Cells in Aspirate (Count, %) <=10% 11-20% 21-30% 31-40% 41-50% 51-60%	107 4 3 0 0 1	93 3.5 2.6 0 0 0.9	54 59 10 5 0 3	41.2 45 7.6 3.8 0 0

Anxiety was found in 19.1% of patients (MGUS: 22% vs. SMM: 17%), while 33.7% had a previous psychiatric disorder which was not different between patients with MGUS vs. SMM. Among those with a history of psychiatric disorders, 78.3% were taking medication, and 21.7% were receiving treatment from a psychiatrist/therapist. The presence of anxiety or a history of psychiatric disorders did not exhibit significant differences between MGUS and SMM patients (p > 0.05)

Logistic regression analysis was employed to investigate risk factors linked to anxiety and nervousness. Initial univariate analysis considered variables such as age, race, sex, diagnosis (SMM vs. MGUS), history of psychiatric disorders, coping mechanisms, education, employment status, marital status, social support, religious affiliations, alcohol/drug usage, chemical exposure, and family cancer history. Patients with a racial classification of ‘other’ were excluded due to a small sample size (n=2). On univariable analysis, age, race, working status, and a previous history of psychiatric disorders emerged as significant for anxiety and nervousness (p = 0.001, 0.033, 0.045, and <0.001, respectively). In a multivariable model encompassing these four significant variables, both race and history of psychiatric disorders retained significance (p = 0.037 and p = 0.006, respectively). White patients exhibited five times higher odds of experiencing anxiety, while those with a history of psychiatric disorders had 2.6 times higher odds (OR = 5.10 and 2.684, respectively). **[Table.3]**

**Table 3.** Logistic Regression univariate and multivariable analysis of different variables with anxiety

	Univariable Regression		Multivariable Regression	
Variable	Odds Ratio	P value	Odds Ratio	P value
Age (Continuous)	.953	.001	.965	0.076
Race (Reference: White)	.203	0.033	.196	0.037
History of Psychiatric Disorder (Reference: No)	3.303	<0.001	2.684	0.006
Working Status Retired (Ref) Working Other	- 2.167 2.737	0.045 .032 .044	- 1.342 2.399	0.373 .531 .163
Sex (Ref: Male)	1.7	.108	-	-
Diagnosis (MGUS/SMM)	.717	.307	-	-
Presence of Coping Mechanism (Reference: No)	.416	.349	-	-
Education Level (Reference: High School)	.739	.799	-	-
Marital Status (Reference: Married)	.682	.367	-	-
Religious Affiliations (Reference: Yes)	1.696	.142	-	-
Alcohol/Drug Usage (Reference: No)	.779	.602	-	-
Exposure to Chemicals (Reference: No)	1.459	.297	-	-
Family History of Cancer (Reference: No)	1.311	.557	-	-
Living Status (Reference: Living with someone)	.402	.148	-	-

A total of 73 quotes documenting patient sentiments regarding their diagnosis were noted by the social worker. These quotes were subjected to analysis and categorized into themes, including fear, stress, and/or anxiety; religious coping; coping through learning; initial emotional reactions; feelings of upset, shock, frustration, and irritability; avoidance and denial; fluctuations in emotions; strategies for coping and dealing with the diagnosis; and the receipt of social support. The most prevalent theme was ‘fear, stress, and/or anxiety,’ encompassing 32 (44%) of the quotes.

Patients’ emotional responses to their diagnoses revealed several overarching themes. Many patients turned to religious coping, placing their trust in faith and acceptance. One patient eloquently stated, “I’m just trusting God. Whatever His will is, I will be satisfied with it.” Anxiety, stress, and fear were commonly reported emotions, with individuals grappling with newfound concerns. As one patient candidly expressed, “I’ve never felt this way before. I don’t know if I’m depressed, but when I start thinking about all of this, it won’t go away. I’m just worrying, what about my kids?” For some, the process of learning about their diagnosis was a source of coping. As one patient revealed, “I have coped with my diagnosis thus far by learning more about my disease and keeping my mind busy.”

Additionally, initial emotional reactions ranged from reluctance and near-fainting to the acknowledgment of the importance of family and grandchildren. Patients spoke of their feelings of being overwhelmed, depressed, and anxious in the face of their diagnoses. Frustration, irritability, and a sense of avoidance were also expressed, highlighting the complex array of emotions experienced. As one patient succinctly put it, “I just don’t like it, but just deal with it.” Importantly, social support played a vital role, with patients receiving solace from family, friends, and faith, as well as through sharing their diagnosis with loved ones. Additional information regarding the number of quotes within each theme, along with specific examples for each theme is available in **Table.4**

**Table 4.** Thematic Analysis of Quotes (n = 73)

Theme	Number of Quotes Under this Theme	Examples of this Theme
Religious Coping	5	“I’m just trusting God. Whatever His will is, I will be satisfied with it.” states this diagnosis "is scary, but I'm hopeful. I have faith in God and I exercise my faith daily."
Fear, Stress, And/Or Anxiety	32	“I’ve never felt this way before. I don’t know if I’m depressed, but when I start thinking about all of this, it won’t go away. I’m just worrying, what about my kids?” "I'm a little worried. I have lost some sleep.”
Coping by Learning	4	“In regards to recent health concerns, pt discussed the process to be “scary”. She discussed looking forward to meeting with her oncologist to learn of her diagnosis and if needed treatment recommendations.” Pt did become tearful during the interview when discussing about MGUS. He states he has coped with his dx thus far by learning more about his dz and keeping his mind busy.
Emotional Initial Reaction	11	“I’ve come to grips with it. I was kicking and screaming when she brought me down here. At first I didn’t think I wanted to know if I have cancer, but then I thought about my grandchildren so I changed my mind.” Pt expressed she almost passed out when she heard the news of her diagnosis of multiple myeloma last month
Upset (Sadness and Distress)	11	“Related to her diagnosis, she reported feeling overwhelmed, depressed, and anxious. She stated she keeps busy to keep from worrying too much about when her diagnosis” “Related to how he is doing emotionally, pt states feeling down, particularly with the recent increase in doctor visits.”
Shock	6	She reported she was "shocked" and "disappointed" when she received her diagnosis. Today she presented as pleasant. He stated, initially, "the diagnosis was a shock, pretty tough." He went on to say it has made him "more involved."
Frustration and Irritability	5	When pt received his diagnosis, he presented with "frustration." He stated he had trouble in the morning getting up and going. He would lie in bed and think of his diagnosis and "get overwhelmed." Not knowing what to expect was scary, she reports anger related to her Dx and Tx
Avoidance and Denial	4	She reported she has not dealt with her cancer diagnosis emotionally. She stated she avoids thinking about because she gets scared if she thinks about it too much. “I just feel like it’s not real”
Fluctuation	9	She reported it has been like a "see saw" since she was diagnosed. "Some days I ignore it and other days I'm overwhelmed." She reported not having a diagnosis was "stressful." She went on to say "one minute I'm feeling okay, but the next I'm stressed."
‘Dealing’ or ‘Coping’	6	He stated he "teared up for a minute" when he was diagnosed, but is dealing with it pretty well. "I just don’t like it, but just deal with it"
Receives Social Support	3	Regarding her emotional state, patient stated that she initially felt shocked and tearful, however, maintains a positive attitude with the assistance of her family, friends, and faith Pt reports learning she may have myeloma has been an emotional roller coaster of anxiety / tearfulness and times of keeping it together. She states after she told her children about this situation that seemed to ease some of her emotional distress

## Discussion

Our study found that one third of patients referred with diagnosis of MGUS or SMM has history of psychiatric disorder. This indicates that pre-existing mental health conditions may significantly influence the psychological response of patients upon receiving a diagnosis of MGUS or SMM.

Patients with MGUS or SMM use various coping strategies, including turning to religion for comfort and meaning, denial to initially protect themselves from distress, and expressing frustration and irritability as natural responses to stress. Seeking social support from family, friends, and support groups is also common and provides emotional validation and practical help. Each strategy offers distinct benefits but also has limitations, with their effectiveness varying based on individual preferences and available resources. Combining these approaches and seeking professional guidance can enhance coping and overall well-being.

The study underscores the importance of adopting a holistic approach to patient care, highlighting the need to address both the medical and psychological dimensions of managing MGUS and SMM. We suggest a comprehensive strategy that includes the identification and management of anxiety as a critical component of patient care. By focusing not only on the medical treatment of these conditions but also on mitigating the psychological impact of anxiety, healthcare providers can significantly enhance patients’ overall well-being and QoL.

Our study has some limitations. The research was conducted at a single medical center, potentially limiting the generalizability of the results to a broader population. Additionally, the reliance on patient self-reporting and social worker interview process may introduce subjectivity and reporting bias, potentially affecting the accuracy of psychosocial assessments. Patient self-report is not inherently a limitation; many argue that direct patient self-report is the most accurate and valid method for collecting data on how patients feel or function. Our study lacks a control arm of individuals with no plasma cell disorder diagnosis. Nevertheless, the study emphasizes the importance of addressing psychosocial well-being as an integral component of care for individuals with MGUS and SMM, as anxiety can significantly impact patients’ overall QoL.

From a practical perspective, we recommend implementing routine anxiety assessments prior to oncologist appointments. By integrating these assessments into regular clinical practice, clinicians can identify anxiety early and provide timely psychological support alongside medical treatment. This proactive approach can enhance overall care by addressing emotional concerns that may otherwise affect patient adherence to treatment plans.

## Conclusions

In conclusion, our study showed about 20% of patients felt anxious about their diagnosis of MGUS or SMM, and around 33% reported previous psychiatric disorder. African American patients were less likely to feel anxious or nervous, while patients with a history of psychiatric disorders were more likely to feel anxious or nervous. Given that anxiety can negatively affect patients’ well-being and quality of life, healthcare providers should prioritize comprehensive care that includes addressing and managing anxiety alongside the medical aspects of the disease. Furthermore, conducting anxiety assessments for patients prior to their oncologist appointments can be advantageous. This practice provides physicians with essential information, facilitating the delivery of optimal care.

### Authorship Contributions

SAH conceived the research idea. JH and HD conducted patient’s interviews. THP and RB collected data. All authors contributed in data analysis, manuscript writing and approval of final version of submission.

### Disclosure of Conflicts of Interest

The authors declare no relevant conflict of interest.

### Data Sharing Statement

Data can be requested from corresponding author, Samer Al Hadidi (salhadidi@uams.edu) for up to one year after publication.
